# Oxidation of Sodium Deoxycholate Catalyzed by Gold Nanoparticles and Chiral Recognition Performances of Bile Salt Micelles

**DOI:** 10.3390/molecules24244508

**Published:** 2019-12-09

**Authors:** Jing Wang, Xu Xu, Hao Chen, Shuai-Shuai Zhang, Yin-Xian Peng

**Affiliations:** School of Environmental and Chemical Engineering, Jiangsu University of Science and Technology, Zhenjiang 212003, China; xuxumada@gmail.com (X.X.); chenhao834950@gmail.com (H.C.); zhang7793622@gmail.com (S.-S.Z.)

**Keywords:** noble metal nanoparticles, catalytical oxidation, chiral recognition, bile salts, supramolecular chemistry

## Abstract

Au nanoparticles (NPs) were prepared by UV light irradiation of a mixed solution of HAuCl_4_ and sodium deoxycholate (NaDC) under alkaline condition, in which NaDC served as both reducing agent and capping agent. The reaction was monitored by circular dichroism (CD) spectra, and it was found that the formed gold NPs could catalyze the oxidation of NaDC. A CD signal at ~283 nm in the UV region was observed for the oxidation product of NaDC. The intensity of the CD signal of the oxidation product was enhanced gradually with the reaction time. Electrospray ionization (ESI) mass spectra and nuclear magnetic resonance (NMR) spectra were carried out to determine the chemical composition of the oxidation product, revealing that NaDC was selectively oxidized to sodium 3-keto-12-hydroxy-cholanate (3-KHC). The chiral discrimination abilities of the micelles of NaDC and its oxidation product, 3-KHC, were investigated by using chiral model molecules *R,S*-1,1′-Binaphthyl-2,2′-diyl hydrogenphosphate (*R,S*-BNDHP). Compared with NaDC, the micelles of 3-KHC displayed higher binding ability to the chiral model molecules. In addition, the difference in binding affinity of 3-KHC micelles towards *R,S*-isomer was observed, and *S*-isomer was shown to preferentially bind to the micelles.

## 1. Introduction

Bile acids are ubiquitous in humans and animals. They are steroid acids biosynthesized from cholesterol in the liver and have important physiological functions. They promote the solubilization of lipids and fat-soluble vitamins in living organisms and facilitate their metabolism [1,2]. Bile acids are usually present in the form of sodium salts in living organisms. Bile salts (BSs) are amphiphilic molecules possessing a rigid steroid backbone with hydrophilic and hydrophobic faces. Due to the unique structural properties, BSs are provided with detergent properties. They are a class of biosurfactants with distinctive aggregation behaviors. The commonly accepted mechanism for the aggregation of BSs suggests that the micellization of BSs takes place in a stepwise fashion [3,4,5]. Primary aggregates with very few BS monomers are formed in aqueous solution driven by hydrophobic interactions between the monomers at low BS concentration. At higher concentration, secondary aggregates characterized by larger aggregation numbers are formed via hydrogen bonding interactions between the primary aggregates. The aggregates of BSs possess hydrophobic pockets that can accommodate hydrophobic molecules, making them ideal for applications in biological science, biochemistry, drug delivery system, and separation science [6,7,8,9,10].

Compared with the conventional surfactants, biosurfactant BSs exhibit exclusive chirality. Chirality is a fundamental phenomenon found in nature and living organisms. Many biomacromolecules, such as proteins, enzymes, nucleic acids, and polysaccharides, which are important foundations of life activities, are homochiral. A pair of enantiomers of chiral molecules may exhibit significant differences in pharmacological activities, metabolic processes, and toxicities [11,12]. Chiral recognition is of considerable significance in life and biological sciences and has aroused widespread concern in the fields of chemistry, medicine, pharmaceuticals, material sciences, and food industries [13,14,15]. The chiral environments of the BS aggregates provide geometrical specificity for chiral recognition. They are widely employed as chiral mobile phase additives for separation of chiral isomers in high performance liquid chromatography (HPLC) and as pseudostationary phases for separation chiral drugs in micellar electrokinetic capillary chromatography (MECC) [16,17,18].

During the past few decades, chromatography [e.g., high performance liquid chromatography (HPLC) and gas chromatography (GC)] and nuclear magnetic resonance (NMR) spectroscopy have been used extensively to identify chiral isomers [19,20]. These conventional methods are not readily amenable to high-throughput screening assays and are unsuitable for real-time analysis. In recent years, some new methods for the determination of the enantiomers have emerged [21,22,23]. Among them, optical methods such as UV-Vis absorption, circular dichroism (CD), and fluorescence have been developed, which can offer direct, fast, reliable, and highly sensitive responses in high-throughput screening assays [24,25,26]. CD spectroscopy can provide information on the absolute configuration of chiral molecules and is most widely utilized to identify enantiomers and their compositions.

**Figure 1:**
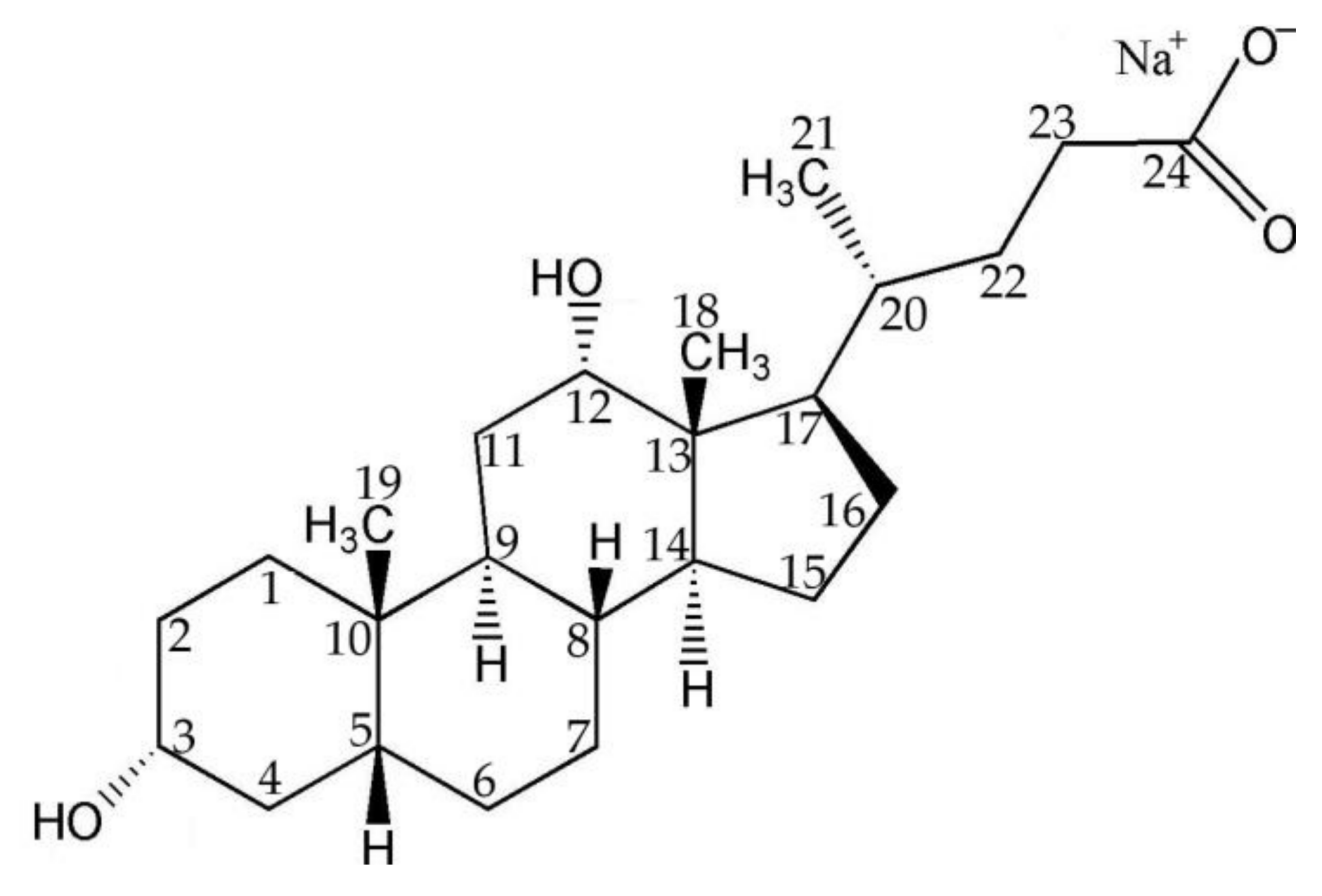
Chemical structure of sodium deoxycholate (NaDC).

Herein, we report the synthesis of Au nanoparticles (NPs) by a photochemical reduction method by using biosurfactant sodium deoxycholate (NaDC) (Figure 1) as reducing agents and capping ligands. It was found that the hydroxyl groups of the BSs were involved in the reduction of the noble metal ions. Under UV light illumination, the hydroxyl groups of NaDC were oxidized to carbonyl groups, and at the same time, Au^3+^ ions were reduced to form Au NPs. The oxidation of hydroxyl groups to carbonyl groups after UV light illumination was confirmed by CD spectrum, revealing a CD signal in the range of 250–325 nm that corresponded to the absorption of carbonyl groups. After finishing the reduction of Au^3+^ ions, the emerging CD signal in the UV region was observed to be further enhanced with the aging time, indicating that more NaDC molecules were oxidized. That is to say, the generated Au NPs acted as catalysts to catalyze the oxidation of NaDC with O_2_. The synthesized oxidation product of NaDC was further exploited to recognize the chiral enantiomers. By monitoring the evolutions of the CD signals and the fluorescence of the chiral enantiomers, the performances of NaDC and its oxidation product in the chiral discrimination were investigated.

## 2. Results and Discussion

### 2.1. Chemical Oxidation of NaDC to 3-KHC Catalyzed by Au NPs

**Figure 2:**
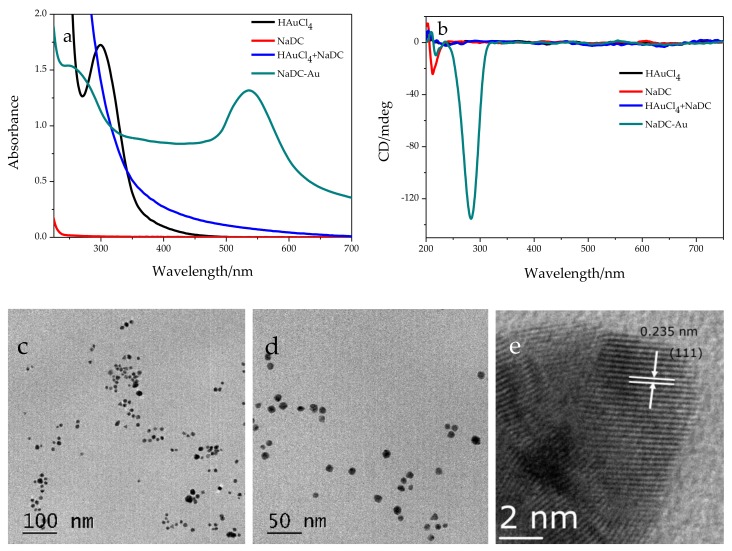
(**a**) Absorption and (**b**) circular dichroism (CD) spectra of HAuCl_4_ solution, NaDC solution, the mixed solution of HAuCl_4_ and NaDC and Au nanoparticles (NPs) prepared by UV light irradiation in the presence of NaDC. (**c**) and (d) Representative TEM images of Au NPs prepared by UV light irradiation in the presence of NaDC. (**e**) High-magnification TEM image of an individual Au NP.

Au nanoparticles were synthesized by mixing aqueous solutions of HAuCl_4_ and sodium deoxycholate (NaDC) under alkaline condition followed by UV light irradiation for 3 h at room temperature. The reaction solution exhibited no absorption in the visible wavelength range before UV light irradiation (Figure 2a). Upon UV light irradiation, the color of the reaction solution changed to red, and an intense absorption peak appeared at ~536 nm, which was the characteristic surface plasmon resonance (SPR) of the Au NPs generated by UV light irradiation of the reaction solution [27,28,29]. Figure 2c‒e shows the typical low- and high-magnification transmission electron microscopy (TEM) images, which illustrate the formation of Au NPs with a diameter of 10.5 ± 2.5 nm after UV light irradiation. The high-resolution TEM (HR-TEM) image displays interplanar spacing of 0.235 nm that corresponded to the (111) planes of face-centered cubic (fcc) Au. The circular dichroism (CD) spectrum showed a strong negative Cotton effect (CE) at about 283 nm, which was different from the CD signal of the NaDC solution at ~212 nm (Figure 2b). It should be noted that the CD signal of NaDC decreased after UV light illumination, suggesting that the concentration of NaDC in the reaction solution decreased. During the photochemical reaction, the NaDC molecules were oxidized; simultaneously, HAuCl_4_ were reduced to gold atoms that aggregated into Au NPs [30,31,32]. It can be inferred that the CD signal appearing at ~283 nm came from the absorption of the oxidation products of NaDC for the carbonyl groups obtained by the oxidation of the hydroxyl groups of the NaDC molecules had an absorption band at ~280 nm.

**Figure 3:**
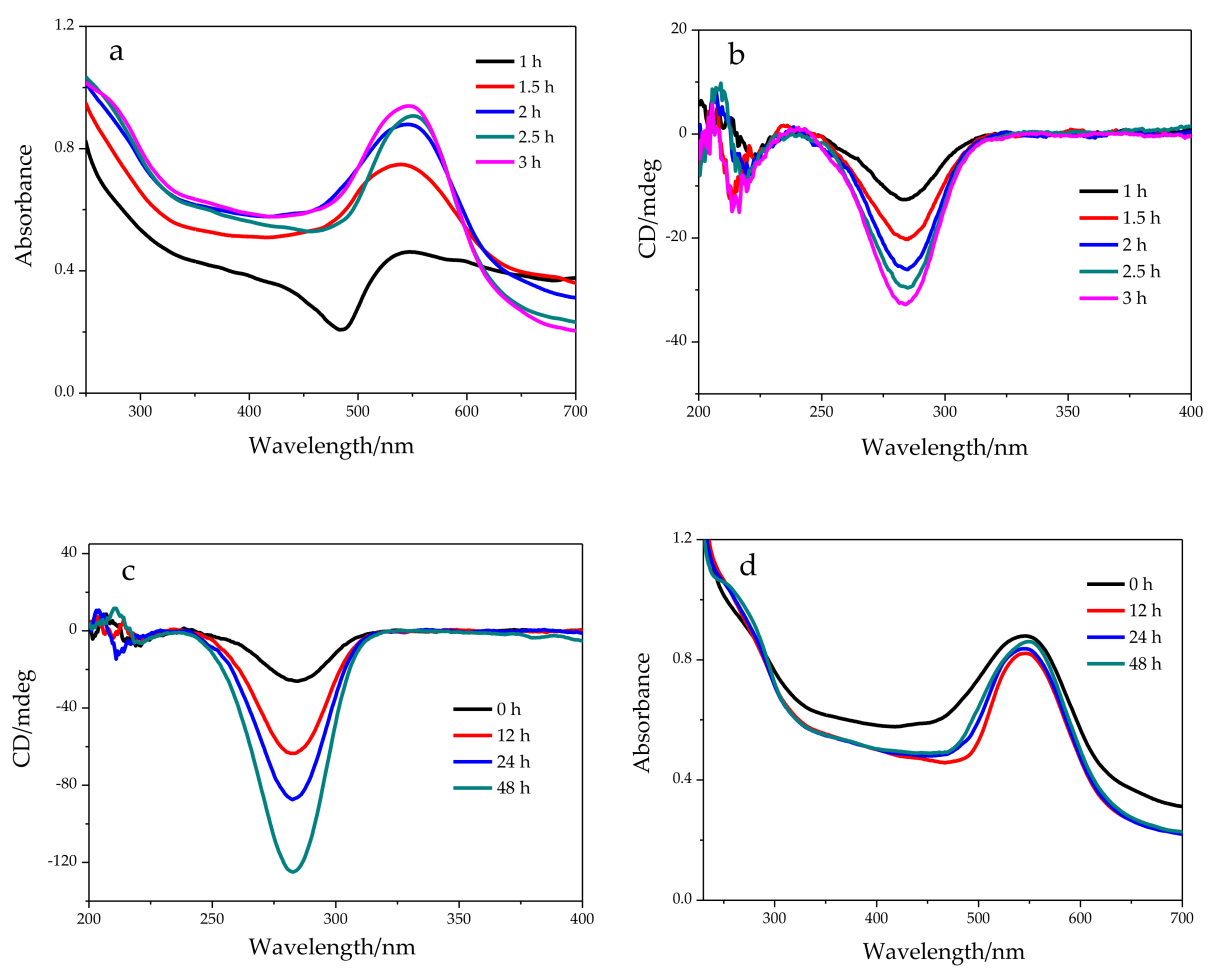
(**a**) Absorption spectra of Au NPs prepared at different times of UV light irradiation. (**b**) CD spectra of the oxidation products obtained at different times of UV light irradiation upon the reaction solutions of HAuCl_4_ and NaDC. (**c**) CD spectra of the oxidation products after aging different times. (**d**) Absorption spectra of Au NPs after aging different times.

The effects of UV exposure times, concentrations, and aging times on the CD signals at ~283 nm were investigated. As shown in Figure 3a, the intensity of the SPR band was found to increase with UV irradiation time, and no significant change in the absorbance was observed after 3 h irradiation, indicating the completion of the reduction of HAuCl_4_. The evolution of CD signal at ~283 nm is shown in Figure 3b, revealing that the CD signal increased gradually with increasing irradiation time. After the completion of the reduction of HAuCl_4_, the sample obtained was aged at 20 °C for different time, and the CD signal was monitored with varied aging time. As shown in Figure 3c, the CD signal was enhanced by increasing aging time, whereas the SPR band of Au NPs remained almost unchanged (Figure 3d). It indicates that the generated Au NPs acted as catalysts for the oxidation of NaDC. Noble metal NPs are widely used as catalysts for oxidation reactions (such as alcohol and CO oxidations) and hydrogenation reactions [33,34]. The reaction temperature has an important influence on the reaction rate. Upon increasing the temperature, the oxidation reaction rate was obviously accelerated (Figure S1).

**Figure 4:**
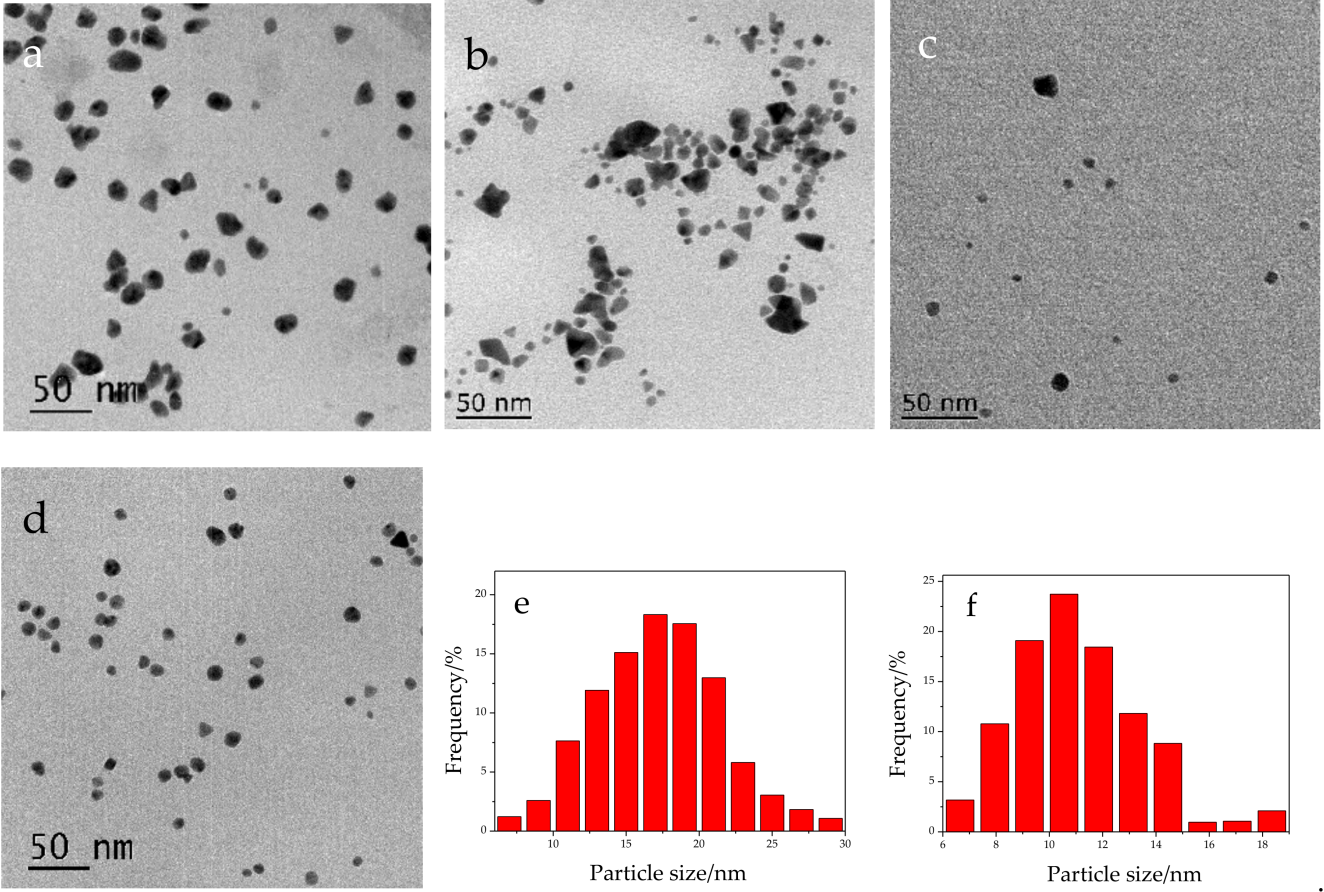
Representative TEM image of Au NPs prepared by UV light irradiation with different concentration of NaDC: (**a**) 3 mM, (**b**) 6 mM, (**c**) 12 mM, and (**d**) 24 mM. (**e**) and (**f**) demonstrate the size distribution of Au NPs corresponding to (**a**) and (**d**), respectively.

To reveal the effect of the concentrations of NaDC on the generated CD signals, Au NPs were synthesized in varied NaDC concentrations. Figure 4a–d shows the TEM images of Au NPs prepared with different concentrations of the NaDC (3, 6, 12, and 24 mM), illustrating that the Au NPs prepared at low NaDC concentration were irregular in shape, and with an increase in NaDC concentration, the prepared Au NPs evolved gradually into nearly spherical shapes. The size distributions of the synthesized Au NPs at NaDC concentrations of 3 and 24 mM are shown in Figure 4e,f, respectively. It reveals that the average diameter of the Au NPs prepared at low NaDC concentration was ~17.5 ± 3.5 nm and at higher NaDC concentration was ~10.5 ± 2.5 nm. With increasing NaDC concentration, the size of Au NPs generated decreased, indicating that a higher concentration of NaDC was beneficial to the growth of Au NPs with regular morphology and smaller size.

**Figure 5:**
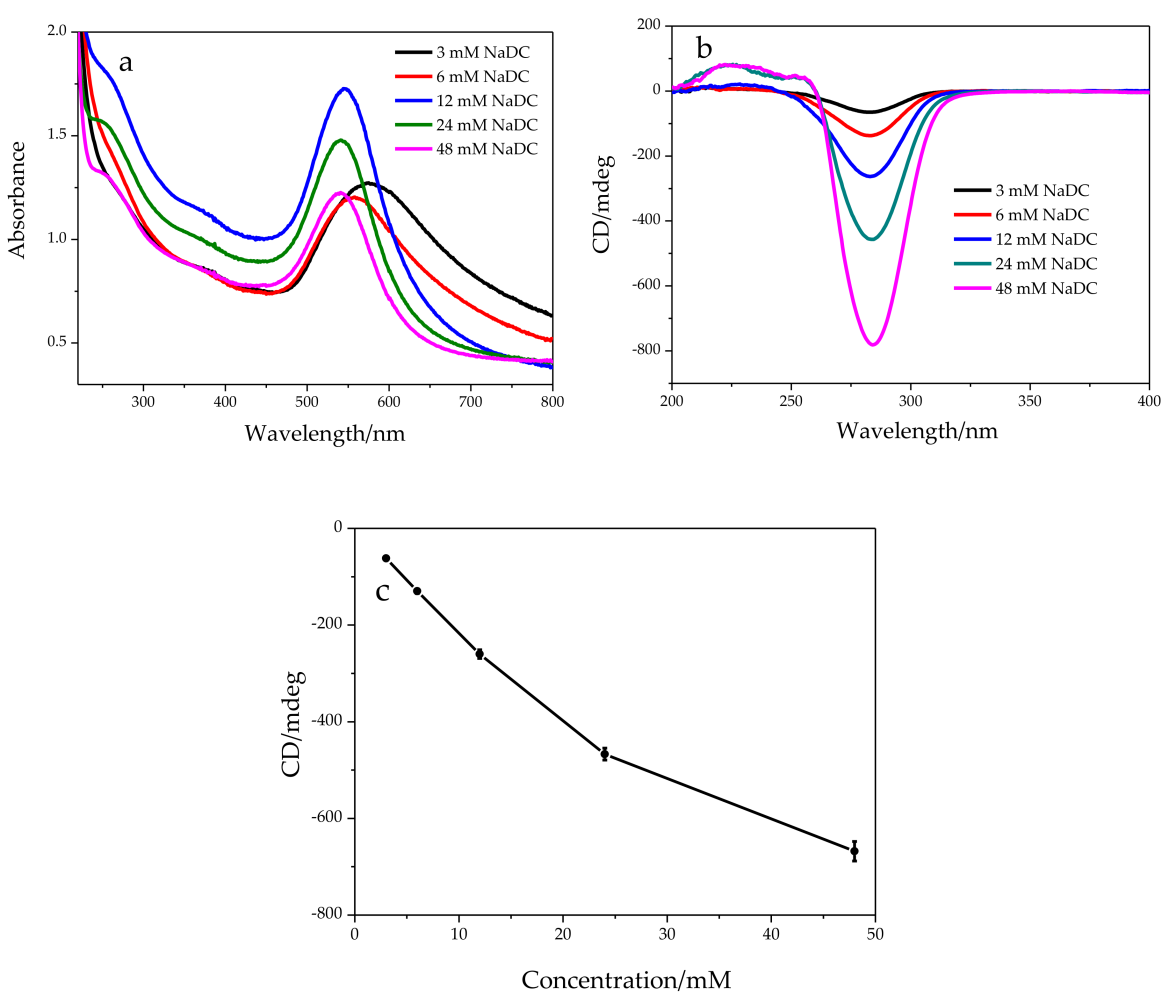
(**a**) Absorption spectra of Au NPs prepared at different concentrations of NaDC. (**b**) CD spectra of the oxidation products obtained at different concentrations of NaDC. (**c**) Relationship between the CD intensity (at 283 nm) and the concentration of NaDC over the range from 3 mM to 48 mM.

Figure 5a exhibits the UV-Vis absorption spectra of Au NPs prepared at varied NaDC concentrations. A blue shift of the SPR band was observed with an increase in the concentration of NaDC, suggesting that the size of the Au NPs decreased, which was confirmed by the results of TEM images. The corresponding CD spectra shown in Figure 5b demonstrate that the CD signals at ~283 nm were strengthened with increasing NaDC concentrations. It can be explained based on the fact that the increase in the concentration of NaDC resulted in an increase in the amounts of oxidized products of NaDC. A nearly linear relationship existed between the intensity of CD signal at 283 nm and the concentration of NaDC when NaDC concentration was below 24 mM, as shown in Figure 5c. This suggests that the catalytical efficiency for the Au NPs prepared at different NaDC concentrations during catalysis of NaDC oxidation was equivalent. When the concentration of NaDC was higher (e.g., 48 mM), the amount of Au NPs was insufficient to catalyze the oxidation of NaDC.

To investigate the effect of particle size on the catalytical conversion efficiency, NaDC powders were added to the solution of Au NPs prepared with 3 mM NaDC to give a total NaDC concentration of 12 mM. The results are shown in Figure S2, revealing that NaDC added after the reduction of Au^3+^ was also oxidized to products by the Au NPs prepared with 3 mM NaDC. The catalytical efficiency of the Au NPs with larger size (prepared with 3 mM NaDC) was ~30% lower than that of the Au NPs with smaller size (prepared with 12 mM NaDC). Furthermore, Au NPs prepared by sodium citrate (SC) reduction were examined in the catalytical oxidation of NaDC, and the results are shown in Figure S3. It can be clearly seen that the catalytical efficiency of SC-capped Au NPs was very low. This may have been due to the presence of the capping agent SC on the surface of Au NPs, which hindered the catalytical oxidation reaction.

**Scheme 1:**
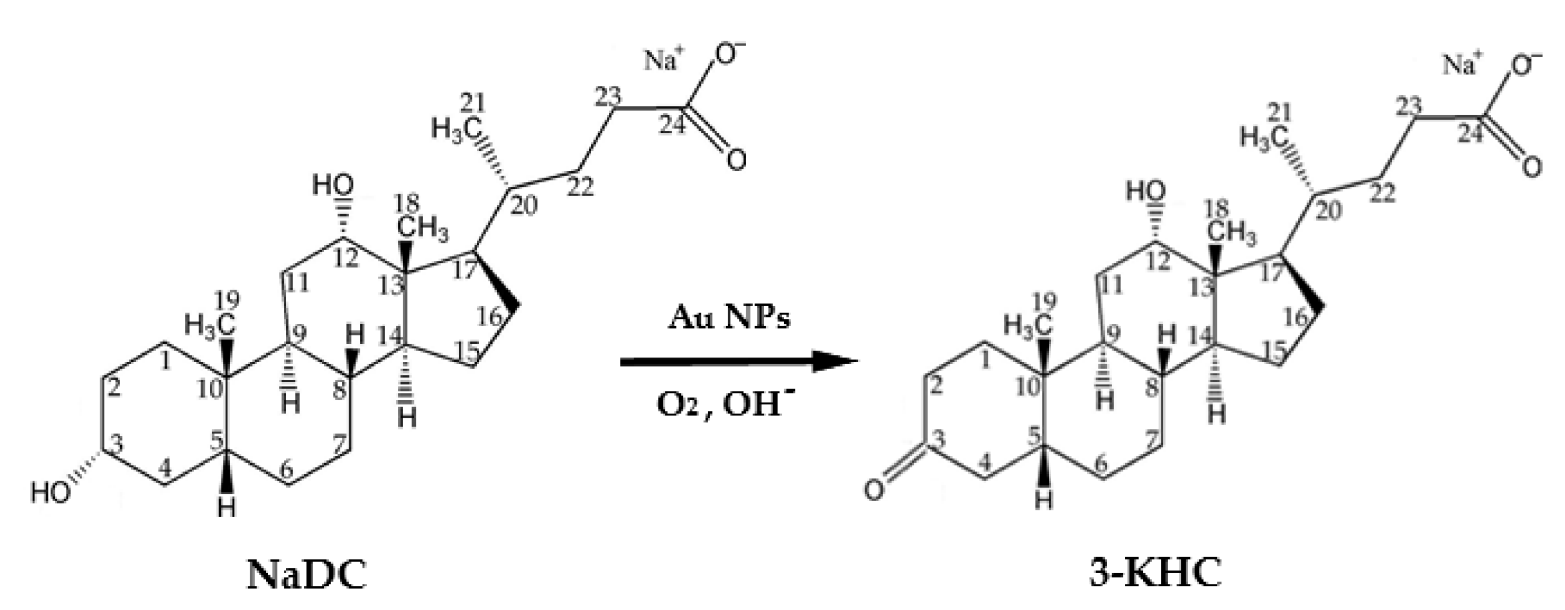
Oxidation of NaDC to sodium 3-keto-12-hydroxy-cholanate (3-KHC) catalyzed by Au NPs.

To characterize the oxidation product of NaDC, mass spectra (MS) and nuclear magnetic resonance (NMR) spectra were measured. ESI mass spectra of the oxidation product is displayed in Figure S4, showing that the molecular ion of the oxidation product was observed at *m*/*z* 389.4. Compared with the molecular ion of deoxycholic acid ([DC]^−^) appearing at *m*/*z* 391.3, the mass spacing 2 Da meant the loss of 2H. Thus, the molecular ion of the oxidation product at *m*/*z* 389.4 could be assigned as [DC-2H]^−^. For NaDC with two hydroxyl groups, the possible oxidation products were 3-keto-12-hydroxy-cholanate, 3-hydroxy-12-keto-cholanate, and 3,12-diketo-cholanate. Based on the results of MS, the oxidation product of NaDC should have been 3-keto-12-hydroxy-cholanate and/or 3-hydroxy-12-keto-cholanate. To further identify the oxidation product, NMR measurement was performed, as shown in Figure S5. The chemical shifts of the two carbon atoms attached to oxygen atoms appeared at 69.97 and 71.15 ppm in the ^13^C NMR spectrum of NaDC. In comparison with NaDC, the ^13^C NMR spectrum of the oxidation product showed the appearance of a signal of carbonyl carbon at 216.18 ppm and the loss of a signal of carbon atom attached to an oxygen atom, suggesting that a hydroxyl group of NaDC was selectively oxidized to a carbonyl group. It can be inferred that the oxidized hydroxyl was at the C3 position, and the product was sodium 3-keto-12-hydroxy-cholanate (3-KHC) (see Scheme 1) by comparison of ^13^C-NMR and ^1^H-NMR data with those reported [35,36,37,38].

### 2.2. Chiral Recognition of R,S-BNDHP in Bile Salt Micelles

**Figure 6:**
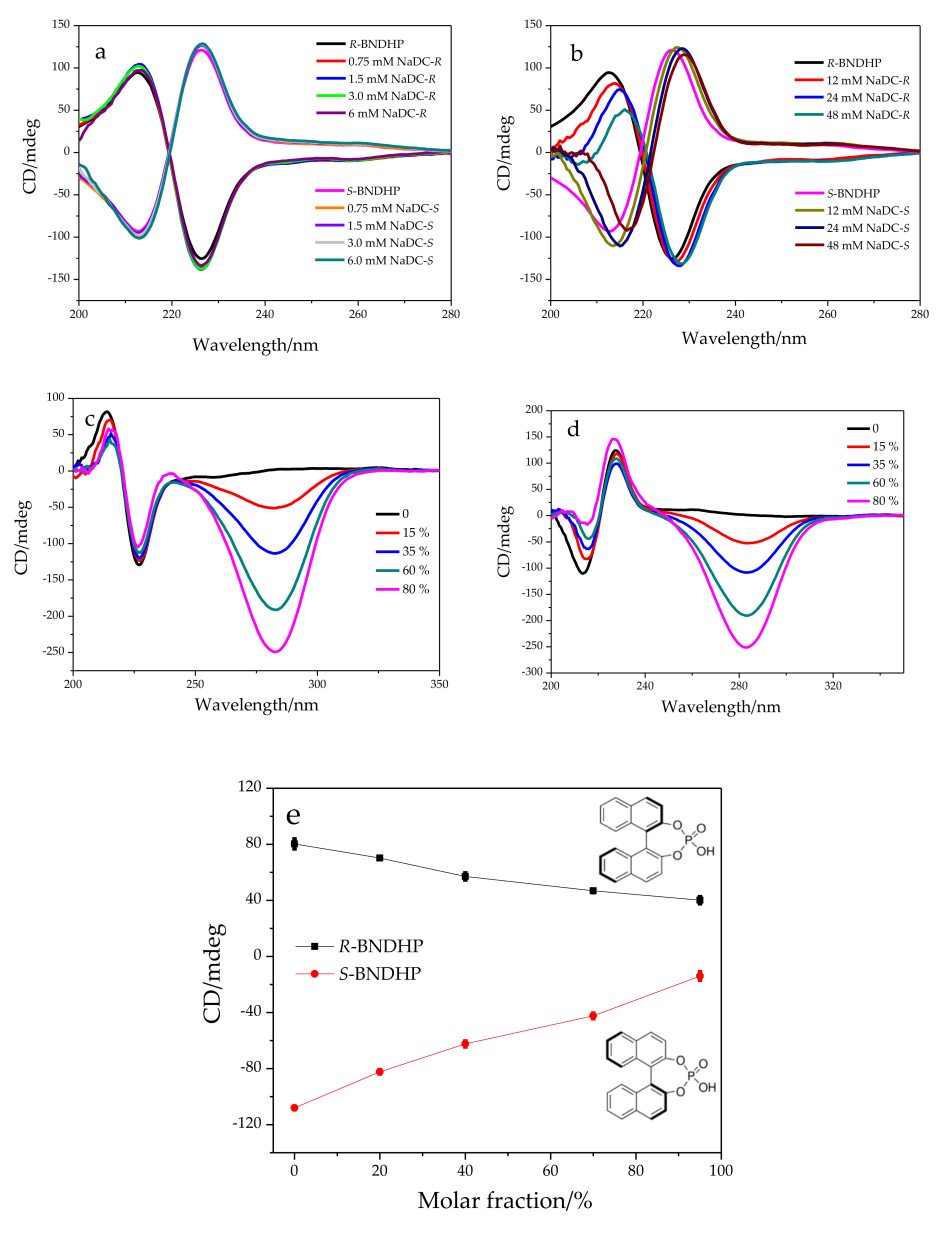
(**a**) CD spectra of *R,S*-1,1′-Binaphthyl-2,2′-diyl hydrogenphosphate (*R,S*-BNDHP) in water and low concentrations of the NaDC solutions. (**b**) CD spectra of *R,S*-BNDHP in water and higher concentrations of the NaDC solutions. (**c**) CD spectra of *R*-BNDHP in water and mixed solutions of NaDC and 3-KHC with a different molar fraction of 3-KHC. (**d**) CD spectra of *S*-BNDHP in water and mixed solutions of NaDC and 3-KHC with a different molar fraction of 3-KHC. (**e**) Comparison of the CD intensity (at 214 nm) for *R,S*-BNDHP in mixed solutions of NaDC and 3-KHC with a different molar fraction of 3-KHC.

Figure 6a shows the CD spectra of *R,S*-1,1′-Binaphthyl-2,2′-diyl hydrogenphosphate (*R,S*-BNDHP) in water and low concentrations of NaDC solutions. The CD spectrum of the aqueous solution of *R,S*-BNDHP showed a split Cotton effect with a mirror image relationship in the region of 200–250 nm, which was assigned to the exciton coupling of ^1^B_b_ transitions of the two naphthyl moieties [39,40]. There were no significant changes in CD signals when *R,S*-BNDHP molecules were dissolved in low concentrations of NaDC solutions. It should be noted that for *R,S*-BNDHP in a higher concentration of NaDC solution the Cotton effect at the short wavelength was gradually weakened (Figure 6b), which meant that the coupling effect of transition dipoles between the two naphthyl moieties was weakened. It is well known that NaDC molecules form micelles at high concentrations, which provides a hydrophobic environment capable of accommodating guest molecules [41,42,43,44,45,46,47]. It is supposed that one of the naphthyl moieties of the BNDHP is embedded in the micelles, which results in the weakening of the coupling between the two naphthyl moieties.

Figure 6c,d exhibit the evolution of the CD spectra of *R,S*-BNDHP in the solutions of NaDC and 3-KHC with different molar fractions. The intensity of the Cotton effect at the short wavelength decreased with an increasing molar fraction of 3-KHC in the mixed micelles, which was accompanied by a red shift of the Cotton effect. These results suggest that the increase in the concentration of 3-KHC facilitated the binding of the *R,S*-BNDHP in the micelles. As shown in Figure 6e, comparison of the change in the intensity of the Cotton effect at the short wavelength for the *R,S*-isomer of BNDHP indicates that there was a considerable difference in the binding ability between *R,S*-BNDHP with the mixed micelles. The observation shows that the *S*-isomer preferred to bind with the mixed micelles. This is consistent with the reported literature [38,39].

**Figure 7:**
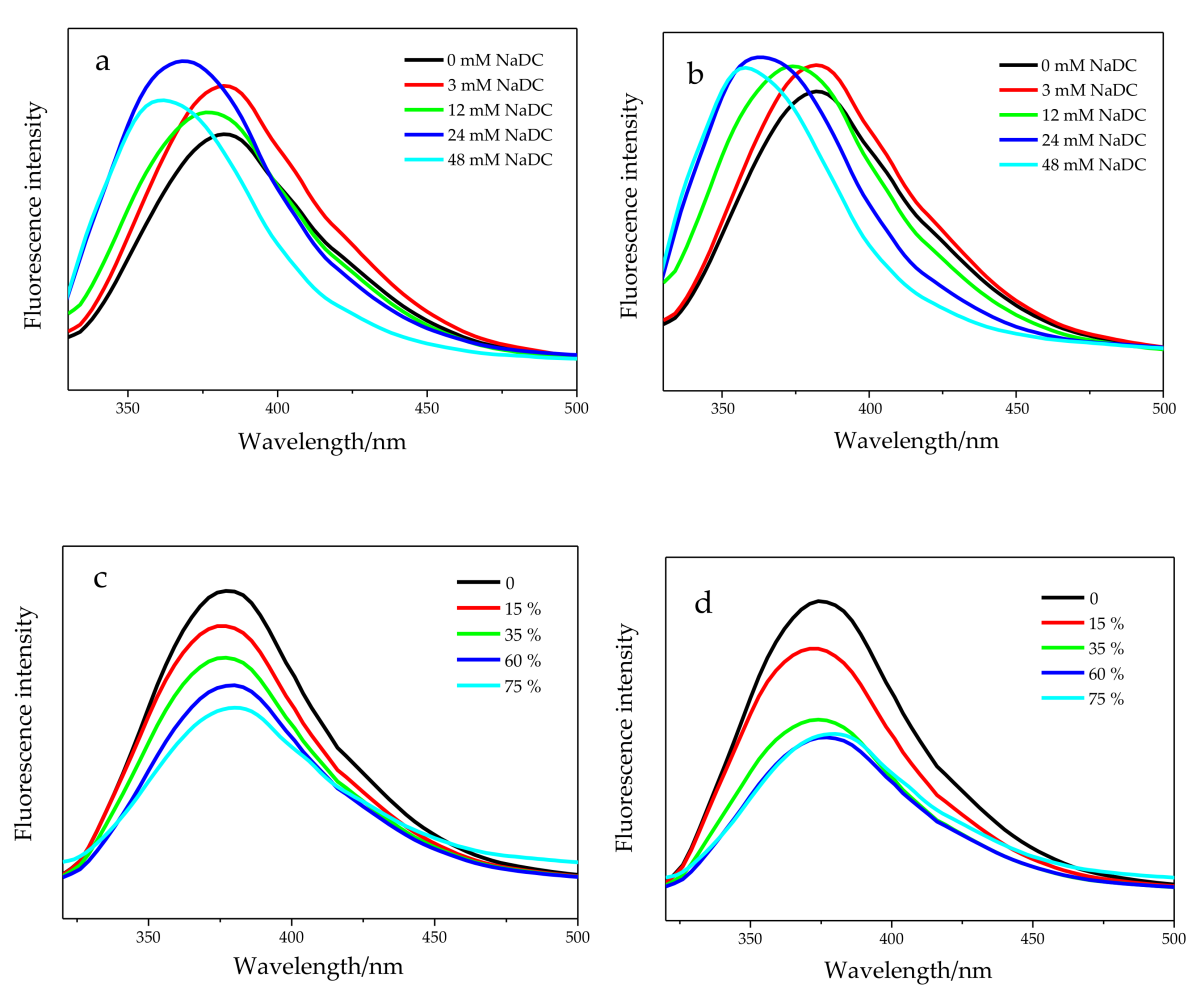
(**a**) Emission spectra of *R*-BNDHP in different concentrations of the NaDC solutions at excitation wavelength of 317 nm. (**b**) Emission spectra of *S*-BNDHP in different concentrations of the NaDC solutions at excitation wavelength of 317 nm. (**c**) Emission spectra of *R*-BNDHP in mixed solutions of NaDC and 3-KHC with a different molar fraction of 3-KHC at excitation wavelength of 305 nm. (**d**) Emission spectra of *S*-BNDHP in mixed solutions of NaDC and 3-KHC with a different molar fraction of 3-KHC at excitation wavelength of 305 nm.

To further understand the interaction between *R,S*-BNDHP and the micelles of NaDC and the mixed micelles of 3-KHC and NaDC, the fluorescence spectra were recorded at excitation wavelengths of 305 and 317 nm. As shown in Figure 7a,b, the emission spectrum of the aqueous solution of *R,S*-BNDHP exhibited a maximum at ~385 nm. In NaDC solutions, the emission spectra of *R,S*-BNDHP were varied with an increase in NaDC concentration. In the low concentration of NaDC solution (0.003 M) below critical micelle concentration (CMC), the emission spectrum of *R,S*-BNDHP was similar to that in the aqueous solution. However, as NaDC concentration increased from 3 to 48 mM, a gradual blue shift of the emission maximum of *R,S*-BNDHP (from 385 to 356 nm) was observed. The significant change in the emission maximum implies that the polarity of the environment around the *R,S*-BNDHP molecules decreased. That is to say, *R,S*-BNDHP molecules were preferentially trapped in the interior of NaDC micelles. The emission spectra of *R,S*-BNDHP in the mixed micelles of 3-KHC and NaDC (the total concentration was 12 mM) are shown in Figure 7c and d. The emission maximum of *R,S*-BNDHP in the mixed micelles was similar to that in the 12 mM NaDC solution (appearing at ~373 nm). With an increase in the molar fraction of 3-KHC in the mixed micelles, the intensity of the emission at ~375 nm decreased gradually. From the results of CD spectra, it is known that the presence of 3-KHC in the micelles facilitated the binding of the *R,S*-BNDHP (Figure 6e). The hydrophobic pocket within the micelles formed by 3-KHC and NaDC could accommodate more than one *R,S*-BNDHP molecule, thus causing the quenching of their fluorescence.

## 3. Materials and Methods

### 3.1. Materials

Hydrogen tetrachloroaurate trihydrate (HAuCl_4_·3H_2_O), sodium deoxycholate (NaDC), deoxycholic acid (HDC), and sodium citrate (SC) were obtained from Sigma-Aldrich (Shanghai, China) and used without further purification. (*R*)-(−)-1,1′-Binaphthyl-2,2′-diyl hydrogenphosphate (*R*-BNDHP) and (*S*)-(+)-1,1′-Binaphthyl-2,2′-diyl hydrogenphosphate (*S*-BNDHP) were purchased from Aladdin Bio-Chem Technology Co., Ltd. (Shanghai, China). NaOH was purchased from Nanjing Chemical Reagent Co., Ltd. (Nanjing, China). Millipore-Q water was obtained from a Milli-Q device (18.2 MΩ).

### 3.2. Preparation of Au NPs in the Presence of NaDC

The synthesis of Au NPs was achieved by photochemical reduction of HAuCl_4_ in the presence of NaDC. In a typical synthesis, 237 µL of HAuCl_4_ solution (25.4 mM) was added to 10 mL of sodium deoxycholate (24 mM). Then, 200 µL of NaOH solution (1 M) was added into the mixed solution of HAuCl_4_ and NaDC. The reduction reaction was initiated by UV light irradiation at 254 nm at room temperature. The color of the reaction solution gradually changed from colorless to purple, which indicated the formation of Au NPs. After UV irradiation for different times (0.5, 1, 1.5, 2, 2.5, and 3 h), the reaction solution was aged at room temperature. The samples with other concentrations of NaDC were prepared in a similar way.

### 3.3. Chiral Recognition of R,S-BNDHP

Au NPs were removed by centrifugation from the reaction solution after aging for different times. Then, 35 μL of 2.5 mM *R,S*-BNDHP was added to 1.8 mL of the supernatant collected, and the mixture was vortex-mixed thoroughly and sonicated for 20 minutes. After overnight aging, the CD spectra of the samples obtained were measured.

### 3.4. Characterization

UV-vis absorption spectra of the prepared gold NPs were recorded using a U-3010 spectrophotometer (Hitachi Ltd., Tokyo, Japan) at room temperature. The CD spectra of the samples as well as *R,S*-BNDHP in aqueous solution and different concentrations of NaDC were measured with a JASCO J-1500 CD (Japan Spectroscopic Co., Ltd., Tokyo, Japan) spectropolarimeter using a quartz cell of 1 cm optical path length. Transmission electron microscopy (TEM) images were acquired on a JEM-200CX (Japan Electron Optics Laboratory Co., Ltd., Tokyo, Japan) electron microscope operating at an accelerating voltage of 200 kV. High resolution TEM (HRTEM) images were acquired on a Tecnai G2 F30 S-Twin (FEI Company, Hillsboro, OR, USA) operating at 300 kV. The sample was dropped onto a carbon-coated copper grid and allowed to dry at room temperature before being analyzed. The particle size analysis was performed using Nano Measurer 1.2 software. The statistics were done over ~300 nanoparticles. Fluorescence measurements were carried out by using a FS5 fluorescence spectrophotometer (Edinburgh instruments Ltd., Livingston, UK). For MS detection, the reaction solution after removal of the Au NPs was acidified with 1 M HCl. After overnight aging, the solution obtained was centrifuged. The collected precipitates were dissolved in dilute NaOH solution and then centrifuged at 12,000 rpm for 30 min. The supernatant was collected and acidified with 1 M HCl. After overnight aging, the solution was centrifuged, and the precipitates were dried at 45 °C. The measurement of MS spectrum was performed on an Agilent 6100 LC-MS spectrometer (Agilent Technologies Inc., Santa Clara, CA, USA) with an electrospray ionization (ESI) source operating in negative ion mode. The nuclear magnetic resonance (NMR) measurements were performed on a Bruker AVANCE II 400 MHz spectrometer (Bruker Corporation, Billerica, MA, USA). NMR spectra were recorded at 25 °C in CD_3_OD with tetramethylsilane (TMS) as an internal standard for ^1^H NMR and CD_3_OD signal (49.00 ppm) for ^13^C NMR.

## 4. Conclusions

Au NPs were prepared by photochemical reduction of HAuCl_4_ in the presence of biosurfactant NaDC. The results of CD spectra showed that the generated Au NPs exhibited excellent catalytic performance in the oxidation of NaDC to its oxidation product sodium 3-keto-12-hydroxy-cholanate (3-KHC). The size of the Au NPs had an effect on the catalytic oxidation efficiency. It was observed that Au NPs with the smaller size showed higher catalytic activity over the large ones. The chiral recognition abilities of the micelles formed by NaDC and its oxidation product 3-KHC were investigated by using *R,S*-BNDHP as model molecules. Compared with NaDC, the micelles of the oxidation product 3-KHC exhibited stronger binding ability to the model molecules. It was found that the 3-KHC micelles showed a preference for the *S*-enantiomer over the *R*-enantiomer of BNDHP. 

## Supplementary Materials

Supplementary Materials can be found online. Figure S1: CD spectra of the oxidation products obtained at different temperatures. Figure S2: Comparison in the CD signals of the oxidation products obtained by using Au NPs prepared with 3 mM NaDC as catalyst before and after the addition of the NaDC powders and by using Au NPs prepared with 12 mM NaDC. Figure S3: (a) CD spectra of the oxidation products obtained by using SC-capped Au NPs as catalyst. (b) Absorption spectra of the SC-capped Au NPs after the addition of NaDC powders. Figure S4: Mass spectra of (a) the oxidation products and (b) deoxycholic acid. Figure S5: ^13^C (a) and ^1^H (b) NMR spectra of the oxidation products. The signals of the starting material were marked by asterisks.
