# Supplementary material for: Oxidation of Sodium Deoxycholate Catalyzed by Gold Nanoparticles and Chiral Recognition Performances of Bile Salt Micelles
Source: Molecules. 2019 Dec 9;24(24):4508. doi: 10.3390/molecules24244508 (PMC6943626; doi:10.3390/molecules24244508)
Supplement: Supplementary file 1 [file molecules-24-04508-s001.pdf]

Supporting Information

Oxidation of Sodium Deoxycholate Catalyzed by Gold Nanoparticles and Chiral Recognition Performances of Bile Salt Micelles

Jing Wang *, Xu Xu, Hao Chen, Shuai-Shuai Zhang and Yin-Xian Peng *

School of Environmental and Chemical Engineering, Jiangsu University of Science and Technology, Zhenjiang 212003, China; xuxumada@gmail.com (X.X.); chenhao834950@gmail.com (H.C.); zhang7793622@gmail.com (S.-S.Z.)

* Correspondence: wangjingalice@just.edu.cn (J.W.); pyxhx@just.edu.cn (Y.-X.P.); Tel.: +86-(511)-8440-1181 (J.W.); +86-(511)-8563-5850 (Y.-X.P.)

Table of Contents

S1 CD spectra of the oxidation products obtained at different temperatures.

S2 Comparison in the CD signals of the oxidation products obtained by using Au NPs prepared with 3 mM NaDC as catalyst before and after the addition of the NaDC powders and by using Au NPs prepared with 12 mM NaDC.

S3 (**a**) CD spectra of the oxidation products obtained by using SC-capped Au NPs as catalyst. (**b**) Absorption spectra of the SC-capped Au NPs after the addition of NaDC powders.

S4 Mass spectra of (**a**) the oxidation products and (**b**) deoxycholic acid.

S5 ^{13}C (**a**) and ^{1}H (**b**) NMR spectra of the oxidation products. The signals of the starting material were marked by asterisks.

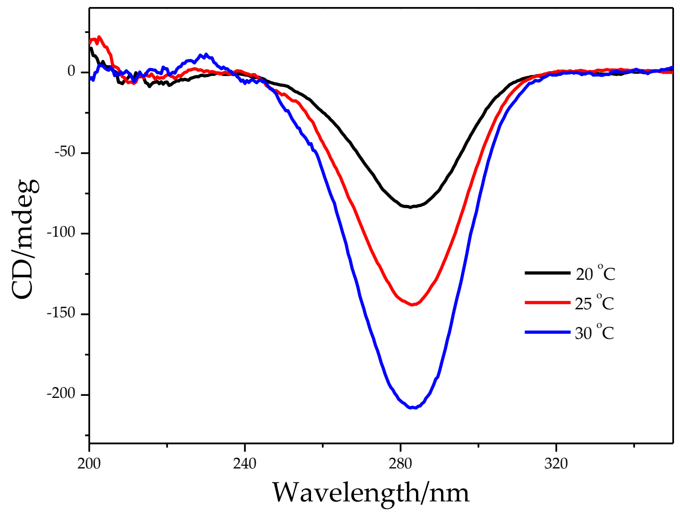

Figure S1. CD spectra of the oxidation products obtained at different temperatures.

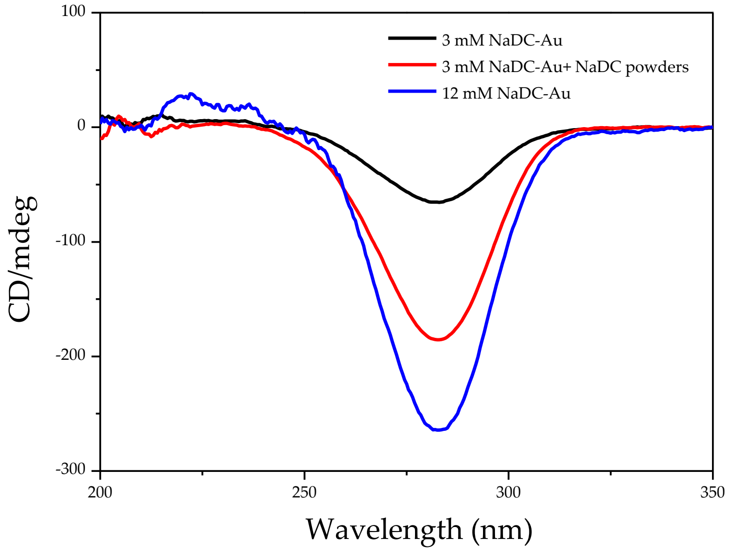

Figure S2. Comparison in the CD signals of the oxidation products obtained by using Au NPs prepared with 3 mM NaDC as catalyst before and after the addition of the NaDC powders and by using Au NPs prepared with 12 mM NaDC.

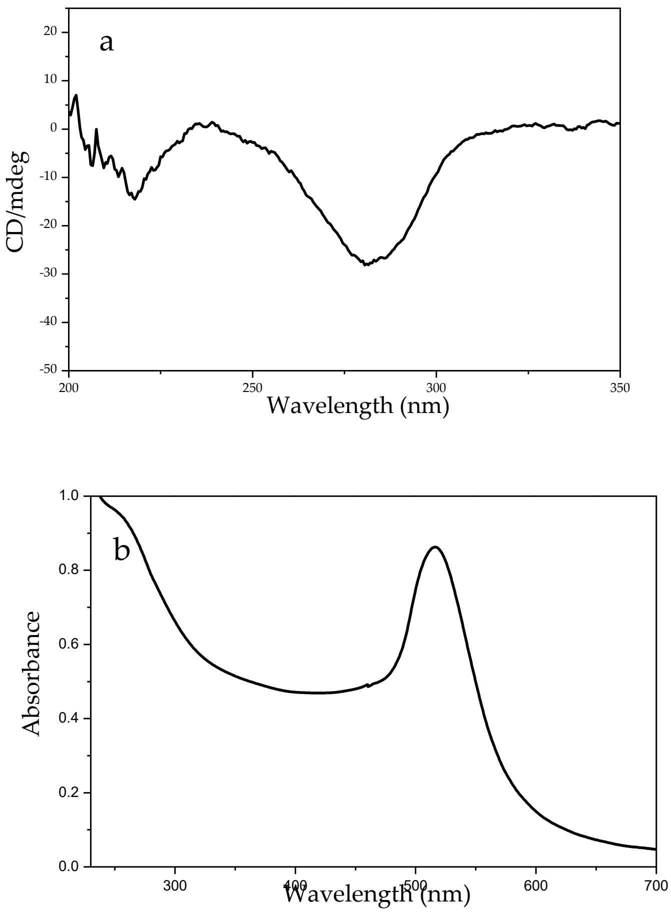

Figure S3. (**a**) CD spectra of the oxidation products obtained by using SC-capped Au NPs as catalyst. (**b**) Absorption spectra of the SC-capped Au NPs after the addition of NaDC powders.

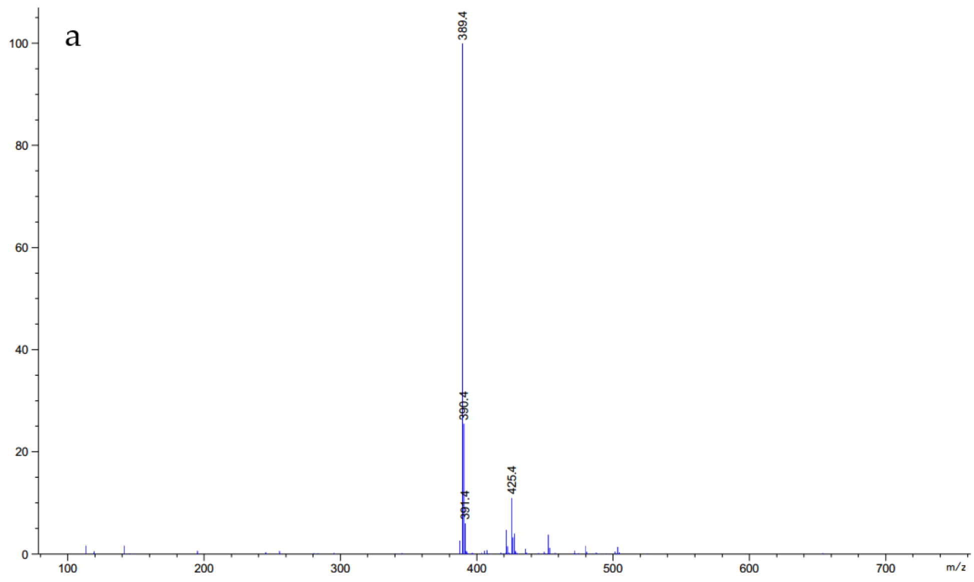

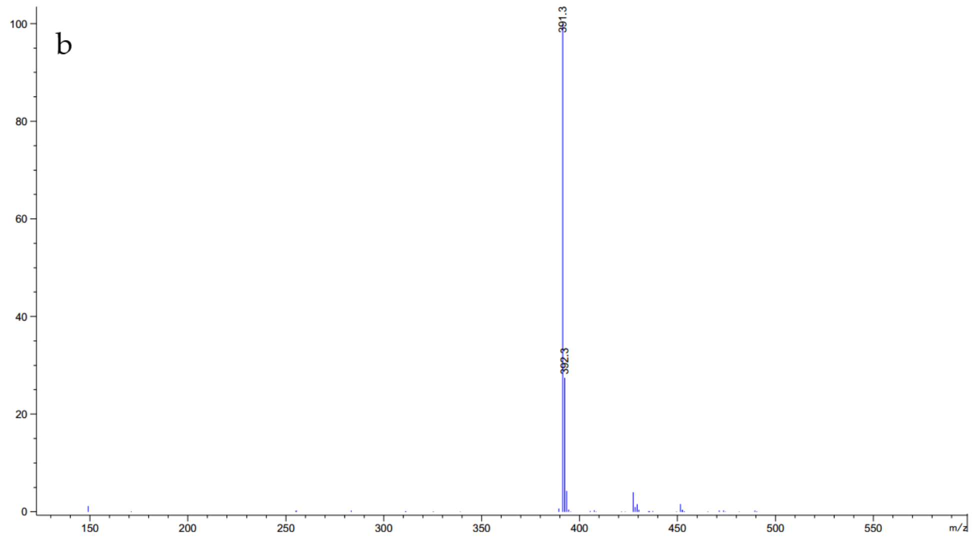

Figure S4. Mass spectra of (**a**) the oxidation products and (**b**) deoxycholic acid.

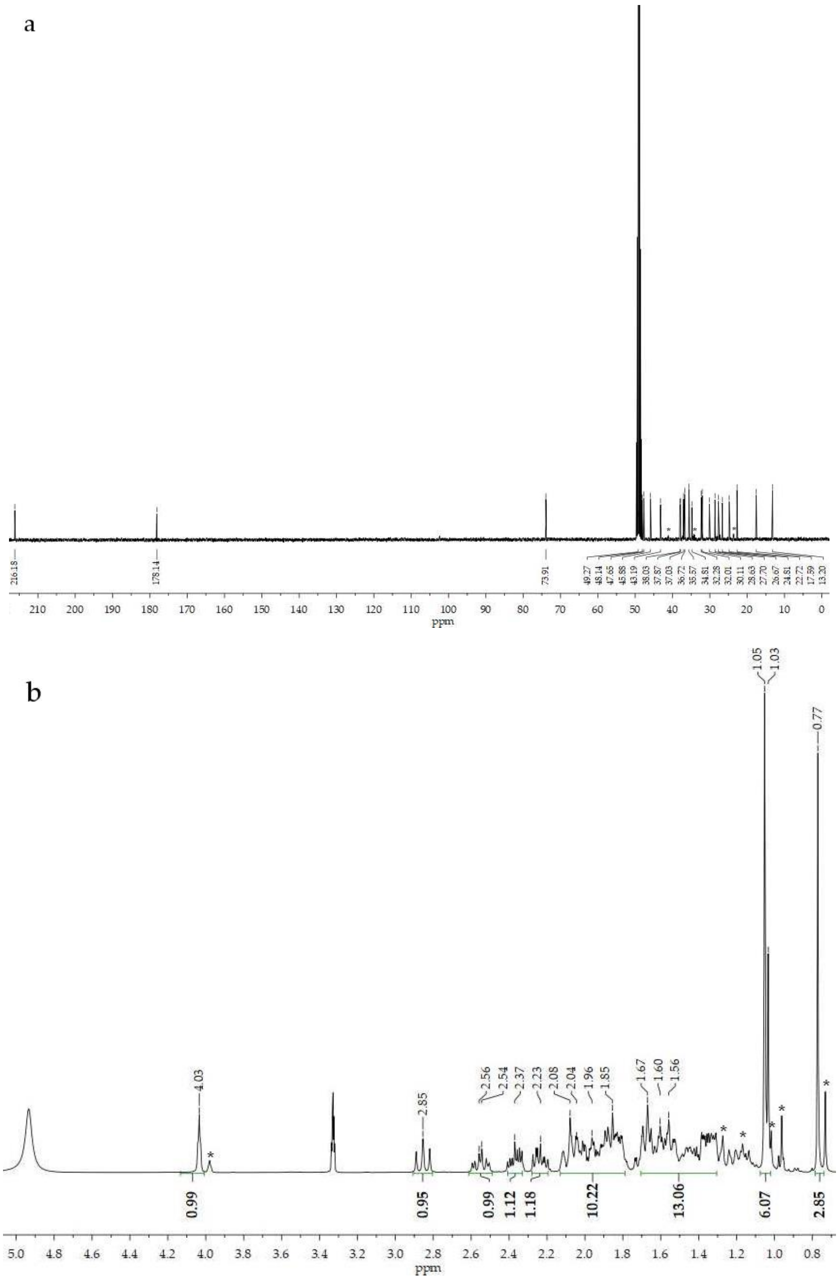

Figure S5. ^{13}C (**a**) and ^{1}H (**b**) NMR spectra of the oxidation products. The signals of the starting material were marked by asterisks.
